# Overcoming Barriers to HIV Prevention and Healthcare Among Sub-Saharan African Migrants in Spain

**DOI:** 10.2196/10478

**Published:** 2018-05-15

**Authors:** Miriam Navarro, Bárbara Navaza, Anne Guionnet, Rogelio López-Vélez

**Affiliations:** ^1^ Infectious Diseases Department National Referral Centre for Tropical Diseases Hospital Universitario Ramón y Cajal Madrid Spain; ^2^ Salud Entre Culturas Madrid Spain

**Keywords:** HIV-prevention, migrants, Africa, access to healthcare, health education

Fakoya and colleagues explored factors associated with access to HIV testing and primary care among migrants living in nine European countries—including Spain—in their study recently published in this journal [1]. The authors highlighted the importance of continued HIV knowledge and awareness initiatives aimed at migrant communities. We would like to emphasize that linguistic and cultural adaptation of such initiatives is crucial to send effective preventative messages and to overcome barriers to healthcare access and medical follow-up, especially among sub-Saharan African migrants (SSAM). Furthermore, we highly recommend the participation of intercultural mediators and we consider that the institutional support is vital to ensure the strategies’ continuity.

We would like to comment on some methods and key results of the HIV prevention program carried out with migrants by the National Referral Centre for Tropical Diseases of the Hospital Ramón y Cajal in Madrid. With the aim of overcoming barriers to healthcare and HIV-prevention among migrants, the program was created in 2006 by a team of physicians, translators, intercultural mediators and a psychologist, focusing on SSAM living in Spain. From 2007, the program (“New citizens, new patients”) started to cover more topics (such as Chagas disease [2], tuberculosis or travel-related diseases [3]) and to reach migrants from different continents and regions (Latin-America and the Caribbean, Eastern Europe, Maghreb, Asia).

This HIV-prevention program—still ongoing—is based on the following pillars:

1. Study and consideration of migrants’ needs and perspectives (qualitative research and KAP—knowledge, attitudes and practices—questionnaires)
2. Design of educational material
3. Community engagement through conduction of:
  - Multilingual seminars in NGOs and migrant associations
  - Public awareness campaigns, street activities and distribution of brochures and condoms on key dates (ie, World AIDS day) and sites (ie, primary healthcare centers)
4. Overcoming barriers to HIV diagnosis and treatment by performing rapid tests and guaranteeing access to medical follow-up
5. Training of intercultural mediators
6. Training of healthcare providers and NGO professionals on HIV prevention with migrants [4]

SSAM participating in our HIV-prevention program have reported to be native-speakers of more than 30 different African languages, Wolof and Bambara being especially prominent. Given this great language diversity, we developed a training program on intercultural mediation for SSAM in 2008 (n=11; eight men and three women). Seven migrant women from other geographical regions were also trained. A second edition of the course was performed in 2014, in which seven of the 19 trainees were SSAM, all men. Currently, they act as interpreters and peer educators in informative seminars performed in several Spanish regions and in medical consultations in Madrid, playing a key role to avoid misunderstandings, overcome linguistic barriers and improve communication among patients and health professionals. Our interpretation and intercultural mediation service obtained official recognition by the Regional Government of Madrid in December 2017.

While coordinating the counseling and intercultural mediation of a pilot program offering rapid HIV testing in seven public primary healthcare centers in Madrid (2010-2013), we reached a significant number of people who had not been tested previously. Uptake of the service by SSAM was remarkable: 7.4% of migrants who used the service (51/687) [5].

Drawing from qualitative and quantitative research, our informative HIV-prevention seminars include key aspects about the functioning of the Spanish health system, as well as specific information for SSAM about blood testing. Previous experiences of SSAM with healthcare systems—both in Africa and Spain—influenced the perception of blood testing as a potentially unhealthy and abusive practice [6]. Reluctance to undergo blood testing was hampering patients’ medical follow-up while increasing barriers for HIV testing and treatment. Throughout our program, we have observed that rapid diagnostic tests—both finger-prick and oral fluid tests—are usually well accepted by SSAM, hence facilitating access to HIV testing. Nevertheless, the abovementioned perceptions of routine blood testing can hamper confirmatory tests and medical follow-up of HIV patients, as we have experienced during medical consultations. For these reasons, we inform about what is done with the blood, and we explain why “so much” blood is sometimes needed, how our body recovers afterwards, how long it takes to get the results and the patients’ rights to be informed about such results. Specific brochures for SSAM were developed, where the abovementioned information about blood testing was included. This material was translated into French, English and Portuguese and is freely available on the Internet, on the websiteof the NGO *Salud Entre Culturas*, working in the Hospital Ramón y Cajal [7]. Our materials are designed by healthcare professionals working along with intercultural mediators and are fully illustrated to reach people regardless of their literacy. In 2017 we performed 150 HIV rapid tests after our seminars with SSAM and three of them (2%) shown a reactive result.

Last, we would like to highlight the importance of measuring the efficacy of the interventions, in order to further adapt them to the changing target population’s profile. Fakoya and colleagues reported additional individual-level obstacles to HIV prevention and testing such as lack of knowledge about HIV and low perception of risk [1]. The existence of HIV was questioned or denied by a significant number of migrants who participated in our program: 13% (487/3759; 2007-2017), being most of them SSAM. Globally, the proportion of participants who believed that HIV existed increased significantly after attending our seminars (47% vs. 95%; n=473; 2008-2011). They also reported a better understanding of HIV and AIDS (n=691; *P*<.001), a decrease on their own discriminatory attitudes towards HIV positive people and an increase in preventative practices such as condom use.  Furthermore, the percentage of participants undergoing HIV testing rose significantly after our intervention (average time between KAPs: four weeks; n=473; *P*<.001).

While analyzing the factors influencing the level of knowledge about HIV using multilingual KAP questionnaires, we observed no correlation between the variable “having received previous information about HIV in Spain” and a higher level of knowledge (2006-2009) [8]. This highlighted the need of better adapting the strategies to reach migrants who did not speak Spanish or were not familiarized with the Spanish healthcare system. Thus, we started to train healthcare providers and NGO professionals on HIV prevention with migrants from 2008 [4].

Working with an interdisciplinary—and highly motivated—team which included migrants acting as intercultural mediators and peer educators was paramount for the effectiveness and acceptability of the HIV prevention program. Another key element for the positive impact of our program was the collaboration with physicians, researchers and experts in infectious diseases working in the Hospital Ramón y Cajal. We would like to conclude by encouraging public institutions to fund interdisciplinary and community-based interventions in order to assure their feasibility and continuous adaptation to the specific needs of the population.
